# Plasma proteomic signature of age in healthy humans

**DOI:** 10.1111/acel.12799

**Published:** 2018-07-11

**Authors:** Toshiko Tanaka, Angelique Biancotto, Ruin Moaddel, Ann Zenobia Moore, Marta Gonzalez‐Freire, Miguel A. Aon, Julián Candia, Pingbo Zhang, Foo Cheung, Giovanna Fantoni, CHI consortium, Katie E. R. Stagliano, Brian Sellers, Yuri Kotliarov, Richard D. Semba, Luigi Ferrucci

**Affiliations:** ^1^ Longitudinal Study Section Translational Gerontology Branch NIA NIH Baltimore Maryland; ^2^ Trans‐NIH Center for Human Immunology, Autoimmunity, and Inflammation NIH Bethesda Maryland; ^3^ Laboratory of Clinical Investigation NIA NIH Baltimore Maryland; ^4^ Laboratory of Cardiovascular Science National Institute on Aging National Institutes of Health Baltimore Maryland; ^5^ Wilmer Eye Institute Johns Hopkins University School of Medicine Baltimore Maryland

**Keywords:** aging, aptamers, healthy aging, humans, plasma, proteomics

## Abstract

To characterize the proteomic signature of chronological age, 1,301 proteins were measured in plasma using the SOMAscan assay (SomaLogic, Boulder, CO, USA) in a population of 240 healthy men and women, 22–93 years old, who were disease‐ and treatment‐free and had no physical and cognitive impairment. Using a *p* ≤ 3.83 × 10^−5^ significance threshold, 197 proteins were positively associated, and 20 proteins were negatively associated with age. Growth differentiation factor 15 (GDF15) had the strongest, positive association with age (GDF15; 0.018 ± 0.001, *p* = 7.49 × 10^−56^). In our sample, GDF15 was not associated with other cardiovascular risk factors such as cholesterol or inflammatory markers. The functional pathways enriched in the 217 age‐associated proteins included blood coagulation, chemokine and inflammatory pathways, axon guidance, peptidase activity, and apoptosis. Using elastic net regression models, we created a proteomic signature of age based on relative concentrations of 76 proteins that highly correlated with chronological age (*r* = 0.94). The generalizability of our findings needs replication in an independent cohort.

## 1 INTRODUCTION

Older age is the main risk factor for a myriad of chronic diseases, and it is invariably associated with progressive loss of function in multiple physiological systems. In some individuals, the combined effect of physiological decline and diseases leads to physical and cognitive disability. Despite its importance for health, most epidemiological research considers aging merely as a confounder, a nuance dimension to be accounted for and then discarded, under the assumption that aging is unavoidable and unchangeable (Fried & Ferrucci, 2016). This view is now changed. As the intrinsic biological mechanism of aging is slowly revealed, there is hope that interventions that slow aging and prevent or delay the onset of chronic disease and functional impairments can be discovered (Kennedy et al., 2014; Lopez‐Otin, Blasco, Partridge, Serrano, & Kroemer, 2013).

A critical goal in the field of aging biomarkers is to identify molecular changes that show robust patterns of change with normal aging, with the assumption that departures from this “signature” pattern provide not only information regarding future risk of pathology and functional decline but also clues on compensatory mechanisms by which our organism counteracts the effects of aging (Sierra, Hadley, Suzman, & Hodes, 2009). Such a signature could be used both to identify individuals in the trajectory of accelerated aging and to track the effectiveness of interventions designed to slowdown biological aging.

A challenge in this field is the need to differentiate between aging and diseases. Most participants enrolled in epidemiological studies include a significant number of individuals affected by pathology or disability, and the proportion of such individuals increase with age. Thus, it is difficult to dissect changes in biomarkers of normal aging from those of disease pathology.

DNA methylation and gene expression have been used to develop molecular markers or signatures associated with chronological age (Bocklandt et al., 2011; Hannum et al., 2013; Horvath, 2013; Lin et al., 2016; Weidner et al., 2014). The “epigenetic clock,” a biomarker index that combines weighted information of a subset of DNA methylation sites raised great interest because it is both strongly associated with chronological age across multiple tissues and populations and independent of age, predicts multiple health outcomes, including cardiovascular disease, cancer, and mortality (Chen et al., 2016; Levine et al., 2015; Perna et al., 2016). These findings suggest that aging is associated with stereotyped and reproducible molecular changes that can potentially be used to identify individuals who are aging faster or slower than the average population. However, the underpinnings of these molecular changes have not been fully elucidated, at least in part because the effect of methylation on DNA function, locally and distally from the methylation site, remains unclear (Declerck & Vanden Berghe, 2018).

A promising alternative to current methods may be to construct a similar aging biomarker clock based on circulating proteins. Proteins are attractive because they directly affect phenotypes and provide direct information on biological pathways that can be involved in many of the physiological and pathological manifestations of aging. However, performing discovery proteomics is challenging because of the wide dynamic range of plasma proteins and because of the interference from large, multiply charged proteins such as albumin, apolipoprotein A1, and C‐reactive protein (Geyer, Holdt, Teupser, & Mann, 2017). Attempts to address this challenge by depletion of highly abundant proteins from plasma samples have generated conflicting results, with some suggestions that proteins in depleted samples are no longer representative of the those in the original sample (Bellei et al., 2011). An alternative approach is to use the SOMAscan assay, a technology that uses slow off‐rate modified aptamers (SOMAmer)‐based capture to quantify multiple proteins in human biological liquids, including plasma (Baird, Westwood, & Lovestone, 2015; Di Narzo et al., 2017; Menni et al., 2015). Previous studies using this approach were conducted in convenience samples originally collected for purposes other than studying aging, and included people affected by multiple diseases (Di Narzo et al., 2017). It is not clear to what extent the results of those studies reflect age independently of disease.

To address this issue, we conducted proteomic analyses using the version of the SOMAscan assay that measured 1,301 proteins in 240 adults aged 22–93 years, free of major chronic diseases, cognitive, and functional impairment. The goal was to identify proteins associated with chronological age avoiding as much as possible the effect of clinically detectable disease, examine their association with several clinical characteristics, and further compare our results to previous proteomic profile analyses that used the same technology. We further constructed a proteomic signature of age to begin exploring to what extent the proteome can predict chronological age.

## 2 RESULTS

### 2.1 Association of proteins with chronological age

**Figure 1:**
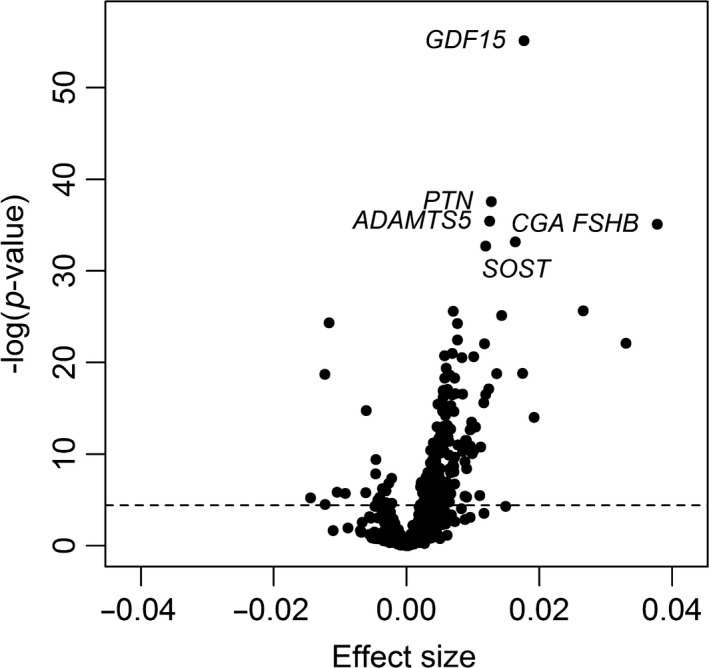
Associations of proteins with age. Volcano plot displaying the association of 1,301 proteins with chronological age. Protein values were log‐transformed and associations with age were tested using a linear model adjusting for sex, race, study (BLSA or GESTALT), and batch. The figure displays the effect size (beta coefficient from the linear model), against significance presented as the −log_10_ (*p*‐value)

**Figure 2:**
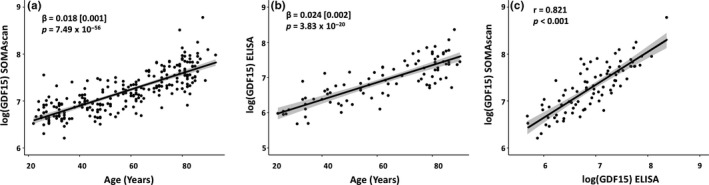
Correlation of GDF15 with age and validation with ELISA assay. (a) The most significant age association was observed for growth differentiation factor 15 (GDF15), which was positively associated with age (*β* = 0.018 ± 0.001, *p* = 7.5 × 10^−56^). To validate association of GDF15 using an independent assay, GDF15 abundance was measured with ELISA on a subset of 88 subjects. (b) GDF15 abundance measured with ELISA correlated with age (*β* = 0.018 ± 0.002, *p* = 3.83 × 10^−20^). (c) Plasma GDF15 measured by ELISA assay was correlated with the measure from SOMAscan, and a correlation of 0.821 was found

Proteomic profiling was conducted on 240 healthy men and women between the ages of 22–93 years. The basic characteristics of the subjects are displayed in Table S1. The association of 1,301 SOMAmers with chronological age was examined. There were 217 proteins (20 negatively associated, 197 positively associated) associated with age (*p* < 3.83 × 10^−5^) in the basic model adjusted for sex, study (Baltimore Longitudinal Study of Aging [BLSA] or Genetic and Epigenetic Signatures of Translational Aging Laboratory Testing [GESTALT]), race, and batch (Tables 1 and S2, Figure 1). Further adjustment for body mass index (BMI), and serum creatinine resulted in 210 (22 negative, 188 positive) age‐associated proteins (Table S2). To explore whether some of the proteins had nonlinear relationship with age, we fitted a model that included an age square term (age^2^) to account for nonlinearity. The proteins were then ranked by the variance explained by the age term for proteins that were linearly correlated with age, or the variance explained by the age plus age^2^ terms for proteins that had evidence of nonlinearity (i.e., had significant age^2^ term). The proteins ranks based on *p*‐values in the linear model were highly correlated with the protein rankings based on a mix of linear and nonlinear models (*r* = 0.96). We concluded that overall, the linear model was adequate for our purpose. The protein with the strongest age association was GDF15 (*β*[*SE*] = 0.018[0.001], *p* = 7.49 × 10^−56^, Figure 2a) that showed positive association with age. To validate the result obtained with GDF15, its plasma level was measured in a subset of 88 subjects using ELISA. GDF15 level assessed by ELISA strongly correlated with age (Figure 2b, *β*[*SE*] = 0.024[0.002], *p* = 3.83 × 10^−20^) confirming the results from the SOMAscan. The correlation between GDF15 abundance measured by the two methods was 0.821 (Figure 2b). Besides GDF15, the top 10 most significant proteins included pleiotrophin (PTN), ADAM metallopeptidase with thrombospondin type 1 motif 5 (ADAMTS5), follicle‐stimulating hormone (FSH; CGA, FSHB), SOST, chordin‐like protein 1 (CHRDL1), natriuretic peptide B (NPPB), EGF‐containing fibulin‐like extracellular matrix protein 1 (FBLN3), matrix metallopeptidase 12 (MMP12), and cathepsin V (CTSV) (Tables 1 and S2).

**Table 1:** Top 10 most significant SOMAmers associated with age

SomaId	Gene ID	UniProt	Target	Model 1^a^			Model 2^b^		
				Beta	*SE*	*p*	Beta	*SE*	*p*
SL003869	GDF15	Q99988	MIC‐1	0.0177	0.0008	7.49E‐56	0.0174	0.0008	6.87E‐55
SL002704	PTN	P21246	PTN	0.0128	0.0008	2.76E‐38	0.0127	0.0008	1.40E‐37
SL004626	ADAMTS5	Q9UNA0	ADAMTS‐5	0.0125	0.0008	3.77E‐36	0.0127	0.0008	6.60E‐36
SL000428	CGA FSHB	P01215 P01225	FSH	0.0378	0.0025	8.17E‐36	0.0377	0.0026	8.15E‐35
SL007631	SOST	Q9BQB4	SOST	0.0164	0.0011	7.00E‐34	0.0162	0.0011	1.32E‐33
SL009400	CHRDL1	Q9BU40	CRDL1	0.0119	0.0008	1.99E‐33	0.0118	0.0008	1.22E‐34
SL002785	NPPB	P16860	N‐terminal pro‐BNP	0.0266	0.0022	2.25E‐26	0.0261	0.0022	7.49E‐26
SL006527	EFEMP1	Q12805	FBLN3	0.0071	0.0006	2.52E‐26	0.0070	0.0006	8.16E‐26
SL000522	MMP12	P39900	MMP‐12	0.0144	0.0012	7.59E‐26	0.0142	0.0012	4.25E‐25
SL006910	CTSV	O60911	Cathepsin V	−0.0116	0.0010	4.61E‐25	−0.0113	0.0010	5.61E‐24

*Notes*

a Model 1: log(SOMAmer)~age + sex + race + study + batch.

b Model 2: Model 1 + BMI + inverse of serum creatinine.

Studies have shown that the number of senescent cells increases with aging in multiple human tissues, including circulating T cells (Liu et al., 2009). Senescent cells are characterized by senescence‐associated secretory phenotype (SASP) that release inflammatory mediators, proteinases and other molecules in the surrounding niche, from where they are eventually released into circulation. Of a list of SASP proteins reported in the literature, 72 were targeted by SOMAmers (Table S3), and 21 of the 72 SASP SOMAmers were significantly associated with chronological age, with an overall significant SASP enrichment (*p* = 0.007; Figure S1).

### 2.2 Proteomic signature of age

**Figure 3:**
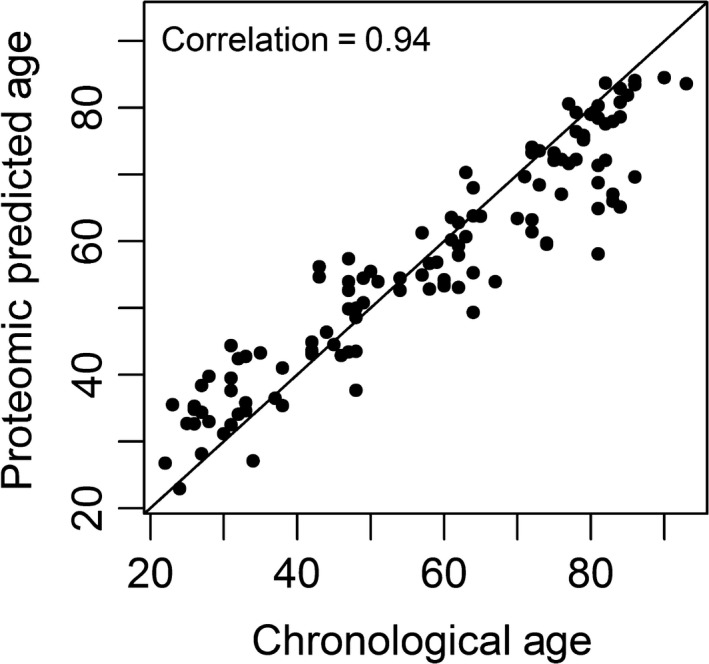
Proteomic signature of age. Using elastic net regression model, proteomic predictors of age were created with variable numbers of predictor proteins in the model. This graphs show the correlation between the predicted age on the *y*‐axis and chronological age on the *x*‐axis for proteomic predictors with 76 predictor proteins. The correlation between predicted age using the proteomic signature and observed age was 0.94

To create a proteomic predictor of chronological age, we fitted an elastic net regression model to select a parsimonious cluster of proteins of the 1,301 proteins measured that best predicted chronological age. We started by randomly splitting the study population into two equally sized groups of 120 participants. The first group was used as a training set and the second as a validation set. From the randomly selected training set of 120 subjects, the elastic net regression selected 76 proteins (Table S4). Of the 76 proteins selected, 37 proteins were among the 217 age‐associated proteins. In the validation set, the correlation between the fitted proteomic age predictor and chronological age was *r* = 0.94 (Figure 3). The correlation between predicted and observed age did not differ by sex (data not shown). To determine the minimum number of proteins required to create a meaningful a proteomic predictor, we fitted a series of models in which we constrained the maximum number of variables to be selected for the calculation of the age predictor in the elastic net regression model (Table 2). This resulted in the generation of predictors with progressively fewer proteins. A total of 13 proteomic age predictors were created ranging from a model with 76 predictor proteins to only one protein, which was the GDF15 (Tables 2 and S4). The precision of the proteomic age predictor was very high even with few proteins, with a correlation of 0.92 between predicted and observed age with as few as 8 proteins. In fact, a predictor including just GDF15 had a relative high correlation with chronological age at *r* = 0.82. The accuracy of the prediction, however, declined substantially when the number of proteins included in the predictor was reduced (Table 2). With the full 76 protein predictor, the mean absolute difference between predicted and observed age was 5.7 years, while the 8‐protein model had a difference of 13.1 years, and the GDF15 only model had a difference of 16.6 years (Table 2). In the 76‐protein age predictor model, only half of the proteins were among significant age‐associated proteins. As the number of proteins included in the predictor decreases, higher percentage of the selected proteins was associated with age, and for predictors with <9 proteins, all the selected proteins were associated with age in univariate analysis.

**Table 2:** Precision and Accuracy of proteomic predictors of age

No. of proteins in model	Correlation between predicted and observed age	% proteins in the predictor associated with age (*N*)	Age_predicted_			|Age_predicted_−Age_observed_|	
			Mean	Min	Max	Mean	*SE*
76	0.943	49 (37)	56.9	22.9	84.5	5.7	4.7
63	0.943	54 (34)	56.9	23.0	83.6	5.9	4.7
58	0.942	57 (33)	56.8	23.3	83.1	6.0	4.7
49	0.942	71 (35)	56.9	25.3	82.4	6.3	4.7
40	0.941	80 (32)	56.9	26.9	82.2	6.7	4.8
27	0.939	93 (25)	56.9	28.5	81.0	7.5	4.9
17	0.936	94 (16)	56.9	33.5	77.5	9.0	5.4
9	0.930	100 (9)	57.0	40.5	72.5	11.4	6.4
8	0.924	100 (8)	57.1	45.2	69.0	13.1	7.1
7	0.872	100 (7)	57.3	50.8	64.7	15.3	8.1
5	0.858	100 (5)	57.3	51.7	63.7	15.6	8.3
3	0.843	100 (3)	57.2	52.6	62.7	16.0	8.5
1	0.815	100 (1)	57.2	54.1	60.8	16.6	8.8

To evaluate how well the proteomic age predictor reflects age, we examined the association of 76 proteins, 8 proteins, and GDF15 proteomic age predictors with 13 age‐associated clinical variables (Table 3). With a few exceptions, all three proteomic age predictors were associated with the clinical parameters in a direction consistent with the associations observed with chronological age. When the associations between proteomic age predictors and clinical parameters were adjusted for chronological age, the associations were no longer significant suggesting that the proteomic age predictors are a good proxy of chronological age.

**Table 3:** Associations of age‐associated clinical parameters with proteomic signatures of age

	Chronological age			76‐protein signature			8‐protein signature			GDF15 signature		
	*b*	*SE*	*p*	*b*	*SE*	*p*	*b*	*SE*	*p*	*b*	*SE*	*p*
IL‐6 (pg/ml)	0.006	0.003	0.037	0.007	0.004	0.063	0.017	0.010	0.093	0.069	0.034	0.044
CRP (μg/ml)	0.012	0.005	0.035	0.012	0.007	0.073	0.039	0.018	0.036	0.185	0.060	0.003
Total Cholesterol (mg/dl)	0.393	0.158	0.014	0.389	0.196	0.049	1.159	0.538	0.033	2.777	1.816	0.129
Glucose (mg/dl)	0.132	0.038	0.001	0.137	0.047	0.005	0.391	0.132	0.004	1.250	0.441	0.005
HBA‐1C	0.008	0.002	4.44E‐06	0.009	0.002	3.25E‐05	0.025	0.006	3.65E‐05	0.077	0.019	1.20E‐04
Blood Urea Nitrogen	0.089	0.018	2.51E‐06	0.120	0.021	1.60E‐07	0.342	0.059	4.84E‐08	0.972	0.204	5.42E‐06
Alkaline Phosphatase	0.207	0.092	0.027	0.169	0.115	0.142	0.545	0.315	0.086	1.946	1.049	0.066
Albumin (g/dl)	−0.007	0.001	2.85E‐06	−0.007	0.002	2.12E‐04	−0.017	0.005	0.001	−0.059	0.016	4.88E‐04
Waist (cm)	0.193	0.047	7.01E‐05	0.185	0.059	0.002	0.518	0.162	0.002	2.147	0.528	8.95E‐05
Grip Strength (kg)	−0.191	0.039	2.67E‐06	−0.216	0.048	1.84E‐05	−0.583	0.133	2.70E‐05	−1.763	0.452	1.63E‐04
Walking speed (m/s)	−0.004	0.001	1.07E‐04	−0.004	0.001	0.005	−0.012	0.004	0.001	−0.047	0.012	1.25E‐04
Systolic Blood Pressure (mmHg)	0.293	0.061	4.17E‐06	0.333	0.075	2.28E‐05	0.813	0.211	1.98E‐04	3.099	0.692	1.80E‐05
Red Blood Cell Distribution Width	0.013	0.003	1.21E‐04	0.013	0.004	0.002	0.036	0.011	0.002	0.084	0.038	0.030

### 2.3 Sex‐specific age associations

**Figure 4:**
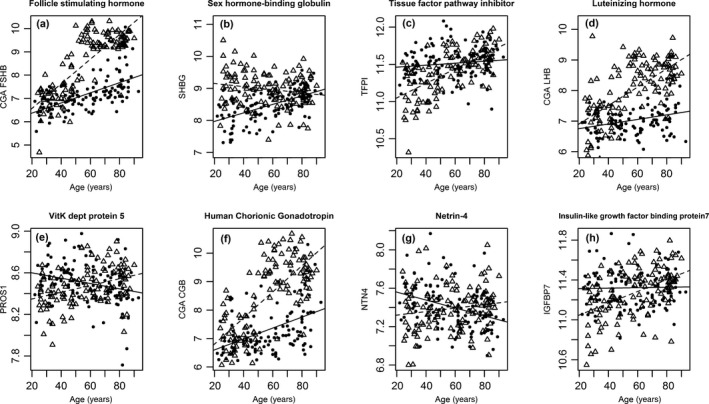
Age‐associated proteins by sex. Association between protein abundance and age differed by sex for eight proteins: (a) Follicle‐stimulating hormone (FSH), (b) sex hormone‐binding globulins (SHBG), (c) tissue factor pathway inhibitor (TFPI), (d) luteinizing hormone (CGA/LHB), (e) vitamin K‐dependent protein 5 (PROS1), (f) human chorionic gonadotropin (CGA/CGB), (g) netrin 4 (NTN4), and (h) insulin‐like growth factor binding protein 7 (IGFBP7). Observations from women are displayed by open triangles and men in closed circles. Regression lines within women (dotted line) and men (solid line) are also displayed

For eight proteins, the correlation with age was different between sexes (Table S4, Figure 4). Not surprisingly, half of these proteins were sex hormones (luteinizing hormone [LH], FSH, human chorionic gonadotropin, sex hormone‐binding globulin [SHGB]). There was a greater positive association of FSH, LH, and SHGB with age in women compared to men (Table S5). There was a positive association of SHGB with age that was significant only in men. The association of tissue factor pathway inhibitor, vitamin K‐dependent protein S, and insulin‐like growth factor binding protein 7 was positively associated with age in women but not significant in men. At last, netrin‐4 had a significant negative association with age in men but not women.

### 2.4 Functional annotation and enrichment analysis

The 217 age‐associated proteins were examined for patterns of functional enrichment. The Kyoto Encyclopedia of Gene and Genomes (KEGG) pathways enriched were “cytokine–cytokine receptor interactions,” “complement coagulation cascades,” and “axon guidance” (Table 4). There were many gene ontology (GO) biological process (30), cellular component (14), and molecular function (12) terms that were enriched among the 217 proteins (Table S6), many of which had shared sets of proteins.

**Table 4:** Top KEGG terms enriched in 217 age‐associated SOMAmers

Term	FDR	Genes
hsa04060:Cytokine–cytokine receptor interaction	5.31E‐10	*IL1R2, CCL3, CXCL9, TNFSF15, TNFRSF8, CCL7, CXCL10, TNFRSF1A, TNFRSF1B, CCL3L1, IL10RA, TNFRSF19, IL15RA, FAS, EGF, IL13RA1, EPO, EGFR, CCL4L1, CCL11, AMH, TNFRSF9, RELT, IFNB1, CXCL16, VEGFA, IL5RA*
hsa04610:Complement and coagulation cascades	9.91E‐07	*PLAT, CD55, FGG, FGA, FGB, SERPINF2, CD59, C6, C5, TFPI, SERPING1, CFD, PLAU, PLAUR*
hsa04360:Axon guidance	2.74E‐04	*NRP1, EFNB3, PLXNB2, EFNA2, EFNB1, EFNB2, NTN1, EPHA1, EPHA2, EPHB2, SEMA6B, SEMA3E, EFNA5, UNC5C, EFNA4*

**Figure 5:**
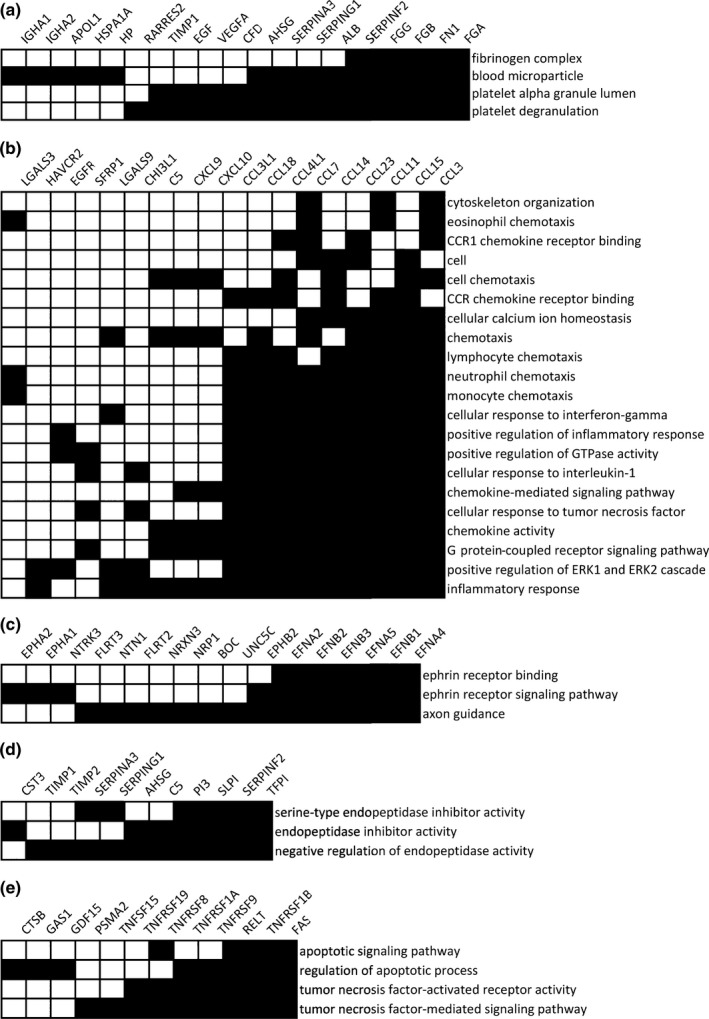
Functional annotation clustering using Database for Annotation, Visualization and Integrated Discovery (DAVID). Pathway enrichment analysis was conducted using DAVID, and to better visualize the shared proteins between the top GO annotation terms, functional annotation clustering was conducted on GO “biological processes,” “molecular function,” and “cellular component” terms. The GO terms and proteins shared among the terms for the top five clusters are displayed

To better understand the patterns of co‐occurrence of proteins, a functional annotation clustering analysis was conducted using DAVID. There were five clusters of GO terms with enrichment scores >3 (Table S7; Figure 5). The first cluster included four GO terms and was defined by 19 proteins (Figure 5a), including blood coagulation proteins. The second cluster comprised 56 proteins and 21 GO terms. The most frequently observed family of proteins in this cluster was the CC chemokines that, together with the other accompanying proteins, represent a protein signature of inflammation and chemokine response (Figure 5b). The third cluster included three GO terms, defined by 17 proteins many of which are ephrin proteins and receptors (Figure 5c). Together with other proteins in the cluster such as netrin proteins, this cluster represents axon guidance. The fourth cluster involved three GO terms, represented by 12 proteins, many of which are implicated in peptidase activity (Figure 5d). The fifth cluster included four GO terms and included 12 proteins. Most of the proteins are members of the TNF receptor family involved in apoptosis (Figure 5e).

## 3 DISCUSSION

In this study, we used the SOMAscan assay to examine the plasma proteomic profile of age in healthy humans. To reduce potential bias from disease and maximize the chance to capture age‐related differences, we selected a sample of individuals spanning a wide age‐range who were very healthy according to strict criteria originally developed for enrollment in the BLSA (Shock et al., 1984). We identified 217 proteins significantly associated with age and show that a precise proteomic predictor of age can be generated using a combination of these proteins. Of the age‐associated proteins, some, such as the GDF15 and NPPB, have previously been described to increase with age, but for many others their association with age has never been previously reported. It is an interesting fact that some of the classic aging biomarkers such as IL6, TNFα, and IGF‐1 were not among the top proteins significantly associated with age. This finding was surprising but may be explained by the exceptional health status of the individuals enrolled in this study. Whether these proteins are better correlated with age in a more representative population that does not exclude persons affected by diseases and disabilities should be explored in future studies.

Several proteomic studies of aging using earlier versions of the SOMAscan platform have been reported. One of these studies was conducted in a sample of women enrolled in the TwinsUK study (Menni et al., 2015). In this study, 1,129 plasma proteins were measured by SOMAscan in 206 women, and the top proteins were tested for replication in 677 subjects from AddNeuroMed, Alzheimer's Research UK, and Dementia Case Registry cohorts. There were 13 age‐associated proteins in the discovery, 10 of which were replicated in the independent samples. In our study, 12 of the 13 proteins that were age‐associated in the TwinsUK study were confirmed to be associated with age. Two other proteomics studies of age were performed in cerebral spinal fluid (CSF) and serum (Baird et al., 2015; Di Narzo et al., 2017). In the first study, 800 proteins were measured in CSF from 90 cognitive normal participants between 21 and 85 years old (Baird et al., 2015). Of these, 248 proteins exhibiting a signal twofold greater than the background were tested for association with age, of which 81 were found to be associated with age. In the second study, 1,128 serum proteins were measured in 88 subjects with ulcerative colitis, 84 subjects with Crohn's disease, and 15 healthy subjects (Di Narzo et al., 2017). There were 130 and 32 age‐associated proteins in patients with ulcerative colitis and patients with Crohn's disease, respectively. It is difficult to directly compare the results from the latter two studies and the present work because of differences in study subjects (healthy vs. disease), biological sample used (plasma vs. CSF and serum), and protein coverage due to the different versions of the SOMAscan used. Due to these differences, less than half of the age‐associated proteins reported in CSF and serum were confirmed in the plasma. It would be of interest to conduct a study examining the proteomic profile in different biological samples within the same individuals to determine whether different proteomic signatures of age differ by sample type.

In our study, we identified many other proteins associated with age that were not previously described using this technology. The most significant age‐associated protein was growth differentiation factor 15 (GDF15), a member of the transforming growth factor‐b cytokine superfamily that plays an essential role in regulating the cellular response to stress signals in cardiovascular diseases and is produced by cardiac myocytes in response to ischemia (Dominguez‐Rodriguez, Abreu‐Gonzalez, & Avanzas, 2011). GDF15 levels are high in animal models with mitochondrial dysfunction, patients affected by mitochondrial disease, and in older than in younger persons, possibly as a response to impaired calcium homeostasis and excessive oxidative stress (Davis, Liang, & Sue, 2016; Fujita, Taniguchi, Shinkai, Tanaka, & Ito, 2016). It is an interesting fact that the increase in GDF15 with aging found in this study is consistent with previous data showing that mitochondrial function decline with aging in humans (Choi et al., 2016). In our cross‐sectional study, the levels of GDF15 were not associated with any cardiovascular disease risk factors including lipids, inflammation markers, blood pressure, and measure of glucose homeostasis. This suggests that GDF15 may not be a strong marker of CVD in exceptionally healthy individuals.

Functional enrichment analysis highlighted some key pathways that are important in aging. The GO term clusters targeted included blood coagulation, chemokine and inflammatory pathways, axon guidance, peptidase activity, and apoptosis. The two main proteins in the blood coagulation cluster were fibrinogen and fibronectin, both previously shown to increase with age, and both related with a pro‐inflammatory state (Folsom et al., 1991; Labat‐Robert, Potazman, Derouette, & Robert, 1981). A second cluster included a number of peptidases, with substantial overlap with the blood coagulation cluster, and included SERPINF2, AHSG, SERPING1, SERPINA3, and TIMP1 suggesting that this second group of proteins taps into some different aspects of blood clotting pathways. Of note, increased levels of all proteins included in the second cluster have been associated with major age‐related conditions. SERPINF2 modulates insulin sensitivity and is associated with cardiovascular diseases and diabetes (Aso et al., 2000; Uitte de Willige et al., 2011). SERPING1 modulates the complement cascade and is important in many inflammatory diseases, including macular degeneration (Ennis et al., 2008). SERPINA3 has been identified as a specific biomarker of delirium and Alzheimer's disease (Padmanabhan, Levy, Dickson, & Potter, 2006; Poljak et al., 2014). TIMP1 has been involved in age‐associated renal sclerotic and impairment kidney angiogenesis (Tan & Liu, 2012). In addition, TIMP1 (together with TIMP3) regulate the extracellular matrix and strongly affect stem cell function and survival (American College of Emergency, 2015; Jackson et al., 2015).

Enrichment analysis also reveals the changes in protein levels of various CC chemokine family. For many of these chemokine proteins, there are reports that aging affect both their gene expression and protein levels (Mo et al., 2003; Whiting et al., 2015; Yung & Mo, 2003). One of these proteins, CCL11 or eotaxin has been proposed as an important factor in neurogenesis in parabiotic models of aging in mice models (Villeda et al., 2011). Our study provides supportive evidence that these class of proteins change with age in healthy older adults.

The main proteins that define the axon guidance cluster are ephrin proteins that are important in axonal growth during development (Fiore & Puschel, 2003). In adults, some ephrin proteins have been implicated in cancer development (Royet et al., 2017). The implication of changes in ephrin proteins in healthy proteins should be investigated further.

Consistent with the hypothesis of increase apoptosis with aging, one of the enriched functional clusters involved several proteins from the TNF receptor superfamily. The TNF receptor superfamily plays an important role in regulating cell fate, not only apoptosis but also proliferation, and morphogenesis (Aggarwal, Gupta, & Kim, 2012). The TNF receptors can be categorized as activating receptors (such as TNFRSF1B) that control the nuclear factor κB and mitogen‐activated protein kinase (MAPK) pathways, and death receptors (such as FAS) that contain a death domain that induces cell death. TNFR1 (TNFRSF1A) has both activating and death receptor functions and can affect cell metabolism, differentiation, and proliferation (Li, Yin, & Wu, 2013). Soluble TNF receptor 1 (TNFRSF1A) and 2 (TNFRSF1B) have been associated with advance age and aging pathologies such as kidney function, fractures, and cognitive performance (Cauley et al., 2016; Gao et al., 2016; Schei et al., 2017). Our study results would suggest that there may be a more coordinate change in the TNF receptor family with age that may be important determinant of healthy aging.

We sought to examine whether there was enrichment of proteins involved in important aging phenomenon that may not be annotated in established databases. There is a growing interest in understanding the role of senescence in aging. It has been hypothesized that many age‐related, degenerative pathologies are driven at least in part by the accumulation of cell senescence (Campisi & Robert, 2014). An elegant study has shown that clearance of senescent cells can delay age‐associated conditions such as cataract, lordokyphosis, muscle mass and function, and increase longevity in mice (Baker et al., 2011). Several studies have documented that senescence cells release a variety of bioactive molecules including interleukins, chemokines, growth factors, secreted proteases, and extracellular matrix components into the extracellular matrix. Although a comprehensive list of SASP proteins is still not available, in our study we found an enrichment of SASP proteins that has been reported in the literature, suggesting that senescence increases with aging even in subjects who remain relatively healthy. It is possible that these blood biomarkers of age may be used to monitor the trajectories of aging.

Using data from multiple proteins, we created a proteomic signature that is tightly correlated with age. It is an interesting fact that the precision of the proteomic age predictor was not compromised by reducing the number of proteins used in the predictor; however, with fewer proteins, there was a substantial decline of accuracy. Our results suggest that there are stereotypical biological changes that occur with aging that are reflected by circulating proteins. Regardless of whether these protein modifications reflect biological aging or track compensatory mechanisms triggered by aging, similarly to the epigenetic clock, our signature accurately predict age. It is critical that the proteomic “signature” of age identified in our analysis be examined in different populations, including samples representative of the general population.

There are several important limitations to this study related to the SOMAscan technology and the study population. First, while this SOMAscan platform assessed 1,301 proteins, this is by no means a comprehensive list of proteins in the plasma. Most likely, there are other key aging proteins missing from this analysis; thus, our results do not comprehensively represent the aging proteome. For example, we observed that most of age‐associated proteins show increased abundance with age. This trend was also observed in the previous study of aging in plasma using the SOMAscan platform (Menni et al., 2015). It is unlikely that this is a biological phenomenon but rather an artificial observation based on the proteins that are targeted by the SOMAmers. Other proteomic aging studies in humans using technology such as two‐dimensional gel electrophoresis (Byerley et al., 2010) or quantitative mass spectrometry (Waldera‐Lupa et al., 2014) showed an equal number of age‐associated proteins that decreased as well as increased with age. Second, the SOMAscan is not an absolute measure of proteins, and therefore, we cannot make comparisons between proteins. Third, the accuracy of the protein specificity revealed by the SOMAscan technology is still controversial (Schafer et al., 2016). While the aptamers are designed to measure proteins in their native confirmation, there is still a possibility of cross‐reactivity for proteins with high similarity. We validated the measure and the age association for our top protein GDF15 by comparing values obtained with the SOMAScan with those obtained by ELISA. Nevertheless, substantial work remains to be done to validate the other proteins. Large aging proteomic studies conducted with different technologies are needed to provide a comprehensive picture of the aging proteome in addition to validate our findings. A substantial step in this field is to overcome the current limitations of LC‐MS approached to study proteomics in plasma to obtain a comprehensive profile in this highly accessible biological fluid. At last, our study involved individuals that were exceptionally healthy, which is an advantage of our approach, but it can also be a limitation. As we have applied the same selection criteria across the age spectrum, it is most likely that the younger and older populations are different. The older subjects in this study are by all accounts healthy agers, while the likelihood of the younger subjects to be as healthy in older age is not guaranteed. In addition, the healthy older subjects in this study are not generalizable to the average American population.

In summary, using a discovery proteomic approach, we identified over 200 proteins that are robustly associated with age. Our findings could provide a window to a new area of investigation with enormous potential. Future studies are needed to replicate and expand our findings in a larger population and, possibly, in representative cohorts that are followed for many years. Under the assumption that the age‐proteomic profile summarizes the biological mechanisms of aging, one could anticipate that such profile would predict many of the aging phenotypes as well as multimorbidity, disability, and death. If future studies show that longitudinal changes in the age‐proteomic profile track the phenotypic manifestations of aging, plasma proteomics may shed light into the biology of aging and contribute to the development of interventions aimed at preventing the burden of disease and disability in older persons.

## 4 EXPERIMENTAL PROCEDURES

### 4.1 Study population

This study was conducted in healthy men and women participating in the BLSA and the GESTALT studies.

The BLSA study is a population‐based study aimed at depicting physiological and functional trajectories with aging and discover factors that affect those trajectories. The study evaluates contributors of healthy aging in persons 20 years old and older (Shock et al., 1984). Starting in 1958, the BLSA study follows participants for life, at intervals from 1 to 4 years, depending on their age. The GESTALT study began in April 2015 and was aimed at discovering new molecular biomarkers of aging in different cell types and develop new phenotypes that are highly age sensitive and can be potentially applied in epidemiological studies of aging. In both BLSA and GESTALT, participants 20 years or older are recruited from the DC/Baltimore metropolitan area, and only if they are considered healthy based on stringent criteria, including absence of any chronic disease (with the exception of controlled hypertension) and cognitive or functional impairment (detailed in Appendix S1). For the GESTALT study, baseline sample were run in the SOMAscan Assay. For the BLSA study, samples collected at times when all healthy criteria were still met were selected. Both studies share the sample protocol for medical assessment and biochemical measurements and were conducted by expert research nurses and physicians. The study protocol for both studies was reviewed and approved by the Internal Review Board of the National Institute for Environmental Health Sciences (NIEHS) and all participants provided written informed consent.

Information about lifestyle factors such as smoking and years of education were assessed by self‐report. Waist circumference, BMI (ratio of weight in kg to square of height in meters), and blood pressure were objectively assessed during a standard medical exam. Grip strength was measured three times on each of the right and left hand. The highest average grip strength was used. Usual gait speed was measured in two trials of a 6‐m walk; the faster time between the two trials was used in the analysis.

Blood tests were performed at a Clinical Laboratory Improvement Amendments certified clinical laboratory at Harbor Hospital, home of the National Institute of Aging (NIA) intramural research program clinical unit. White blood cell count and red blood cell distribution width was measured as part of the standard CBC using SYSMEX SE‐2100 (Sysmex, Kobe, Japan). Albumin was measured using dye binding BCG, blood urea nitrogen with diazo coupling, total cholesterol, alkaline phosphatase, creatinine with enzymatic methods, HDL and LDL with dextran magnetic, triglycerides with colorimetric methods, glucose with glucose oxidase using the Vitros system (Ortho Clinical Diagnostics, Raritan, NJ, USA). Serum inflammatory markers IL6 (R&D System, Minneapolis, MN, USA) and CRP (Alpco, Salem, NH, USA) were measured with enzyme‐linked immunosorbent assay (ELISA). HbA1C levels were measured using liquid chromatography by an automated DiaSTAT analyzer (Bio‐Rad, Oakland, CA, USA). In a subset of 88 subjects, plasma GDF15 was measured using Quantikine ELISA (Human GDF‐15; R&D Systems).

### 4.2 Proteomic assessment

Proteomic profiles for 1,322 SOMAmers were assessed using the 1.3K SOMAscan Assay at the Trans‐NIH Center for Human Immunology and Autoimmunity, and Inflammation (CHI), National Institute of Allergy and Infectious Disease, National Institutes of Health (Bethesda, MD, USA). The 1,322 SOMAmer Reagents, 12 hybridization controls and 4 viral proteins (HPV type 16, HPV type 18, isolate BEN, isolate LW123), and 5 SOMAmers that were reported to be nonspecific (P05186; ALPL, P09871; C1S, Q14126; DSG2, Q93038; TNFRSF25, Q9NQC3; RTN4) were removed leaving 1,301 SOMAmer Reagents in the final analysis. There are 46 SOMAmer Reagents that target multicomplex proteins of 2 or more unique proteins. There are 49 uniprot IDs that are measured by more than one SOMAmer Reagent. Thus, the 1,301 SOMAmer Reagents target 1,297 Uniprot IDs. Of note, there are four proteins in the final protein panel that are rat homologues (P05413; FABP3, P48788; TINNI2, P19429; TINNI3, P01160; NPPA) of human proteins.

The experimental process for proteomic assessment and data normalization has been previously described (Candia et al., 2017). The data reported are SOMAmer Reagent abundance in relative fluorescence units (RFU). The abundance of the SOMAmer Reagent represents a surrogate of protein concentration in the plasma sample.

Data normalization was conducted in three stages. First, hybridization control normalization removes individual sample variance on the basis of signaling differences between microarray or Agilent scanner. Second, median signal normalization removes intersample differences within a plate due to technical differences such as pipetting variation. At last, calibration normalization removes variance across assay runs. Further, there is an additional interplate normalization process that utilizes CHI calibrator that allows normalization across all experiments conducted at CHI laboratory (Candia et al., 2017). A interactive Shiny web tool was used during the CHI QC process (Cheung et al., 2017).

### 4.3 Statistical analysis

Protein RFU values were natural log‐transformed and outliers outside 4 *SD* were removed. Association of each protein with chronological age was assessed using linear regression adjusted for sex, study (BLSA or GESTALT), plate ID, and race (white, black, other). A second model was examined with further adjustments for white blood cell counts, BMI, and creatinine. To test for differences in age–protein association by sex, an age by sex interaction term was included in the base model. A Bonferroni corrected *p*‐value of 3.84 × 10^−5^ (0.05/1301) was considered significant for the analysis of 1,301 SOMAmer Reagents. To test for potential nonlinear relationship of protein with age, a quadratic (age^2^) term was included in the model. This was carried out by running three regression models. Model 1 is a base model of proteins predicted by covariates (sex, study, plate ID, and race). Model 2 is a model of protein predicted by age and covariates in model 1. Model 3 is a model of protein predicted by age and age^2^ with covariates from model 1. The proteins were then re‐ranked based on variance explained as follows: (a) If the coefficient for age^2^ was not significant (*p* > 0.05), then the variance explained was the differences between adjusted *R* ^2^ from model 2 and model 1; (b) if the coefficient for age^2^ was significant, the variance explained was the difference between adjusted *R* ^2^ from model 3 and model 1.

To determine enrichment of cell senescence proteins, a list of SASP proteins were compiled based on prior research (Coppe, Desprez, Krtolica, & Campisi, 2010; Coppe et al., 2008; Lasry & Ben‐Neriah, 2015). There were 72 unique SOMAmer Reagents that recognized proteins previously reported as SASP proteins. Significant enrichment of SASP proteins among age‐associated proteins was determined using a Fisher's exact test.

### 4.4 Proteomic signature of age

To construct a proteomic age predictor, a penalized regression model was implemented using the R package glmnet. First, a training set was selected by stratified random sampling method selecting 24 subjects from each of the 15‐year age strata (20–35, 35–50, 50–65, 65–80, 80+ years). The remaining 120 subjects were used as a validation sample. In the training dataset, chronological age was regressed on 1,301 log‐transformed protein abundances. The alpha value was set to 0.5 (for elastic net regression) and a lambda of 0.8767859 was selected using a 10‐fold cross‐validation on the training set using the cv.glmnet function. The resulting age‐prediction model from the penalized regression was applied to the validation data and the correlation between predicted and chronological age was examined.

Proteomic age predictor with varying number of predictor proteins was created to explore the minimum number of proteins needed to create a meaningful age predictor. This was carried out by constraining the maximum number of variables selected using the *dfmax* option in the training set. A total of 12 additional age predictors were created with 63, 58, 49, 40, 27, 17, 9, 8, 7, 5, 3, or 1 proteins in the model. These age predictor models were applied to the validation dataset to check for the correlation between predicted and chronological age.

The association between the 13 proteomic age predictors with 13 age‐associated clinical phenotypes (IL‐6, CRP, total cholesterol, fasting glucose, HBA1C, blood urea nitrogen, alkaline phosphatase, serum albumin, waist circumference, grip strength, usual walking speed, systolic blood pressure, and red blood cell distribution width) was tested using multiple linear regression adjusting for chronological sex, study (BLSA or GESTALT), and race (white, black, other). Two clinical variables (IL‐6 and CRP) were natural log‐transformed to achieve near normality. For this analysis, a *p*‐value <0.05 was considered as statistically significant.

### 4.5 Functional annotation and enrichment analysis

To explore whether certain biological processes, molecular function or cellular components are enriched among the proteins that were found significantly correlated with age, a gene enrichment analysis was run on the 217 age‐associated proteins using the Database for Annotation, Visualization and Integrated Discovery (DAVID https://david.ncifcrf.gov/) tool (Dennis et al., 2003). The GO, KEGG pathway enrichment, and functional annotation clustering (Huang et al., 2007) were conducted using default DAVID parameters. Enriched GO and KEGG pathways were considered significant at FDR or Benjamini–Hochberg corrected *p* < 0.05.

## DATA AVAILABILITY

Data proteomic data generated from this study are available upon request. Please contact the corresponding author for further information.

## CONFLICT OF INTEREST

B.S. is a former SomaLogic, Inc. (Boulder, CO, USA) employee and a company shareholder. The remaining authors have no competing interests to declare.

## AUTHOR CONTRIBUTIONS

LF directed and supervised the project. SOMAscan assay was run by AB and GF and supervised by KS, BS, and YK. JC, FC, BS conducted proteomic data normalization and cleaning. TT, AZM, and MA conducted the statistical and bioinformatic analysis. RM and RDS contributed to the interpretation of data. TT prepared the manuscript and all authors have contributed to and approved the final version of the manuscript.

## APPENDIX 1

### MEMBERS OF THE CHI CONSORTIUM

#### 1

Katie E. R. Stagliano^1^, Brian Sellers^1^ and Yuri Kotliarov^1^

^1^Trans‐NIH Center for Human Immunology, Autoimmunity, and Inflammation, NIH, Bethesda, MD, USA
