# Supplementary material for: Plasma proteomic signature of age in healthy humans
Source: Aging Cell. 2018 Jul 11;17(5):e12799. doi: 10.1111/acel.12799 (PMC6156492; doi:10.1111/acel.12799)
Supplement: Supplementary file 1 [file ACEL-17-e12799-s001.docx]

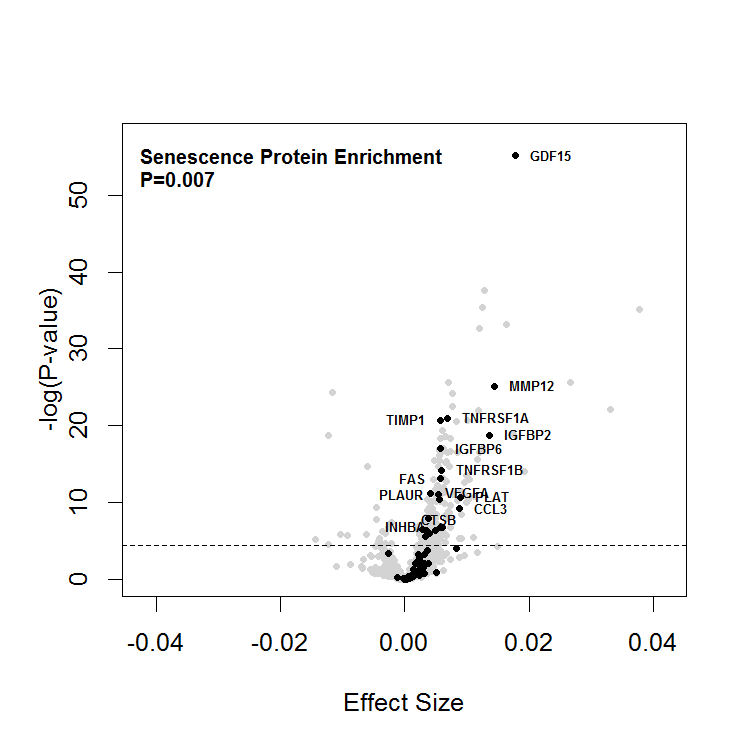


**Supplemental Figure 1** – Volcano plot of age associations for SOMAmer that target senescence- associated secretory phenotype (SASP) proteins.
